# Prevalence of dementia and associated factors in older people's users of primary health care in Brazil

**DOI:** 10.1002/alz70860_099855

**Published:** 2025-12-23

**Authors:** Murilo Bastos, Fernando Sluchensci dos Santos, Michael Pereira Da Silva, Iria Barbara de Oliveira, Weber Cláudio F. N. da Silva, Juliana Sartori Bonini

**Affiliations:** ^1^ State University of the Midwest, Guarapuava, Paraná, Brazil; ^2^ Federal University of Rio Grande, Rio Grande, Rio Grande do Sul, Brazil; ^3^ Universidade Estadual do Centro‐Oeste, Guarapuava, Paraná, Brazil

## Abstract

**Background:**

The aging population faces growing challenges from the increasing prevalence of chronic diseases. Dementia, one of these diseases, is an umbrella term for many forms of dementia, and Alzheimer's Disease is the most prevalent. In addition, there is no cure for dementia and pharmacological therapy has limited results, which brings attention to prevention, especially in many populations and risk factors. Thus, this study aimed to screen for dementia and associated factors in older people attending on primary health care services in a municipality in Paraná, Brazil.

**Method:**

Secondary data from a dementia screening initiative conducted in 2023 across 14 basic health units in a municipality in southern Brazil was utilized. Information was collected on the prevalence of dementia using the Clinical Dementia Rating (CDR) and associated factors, including sociodemographic and behavioral variables and comorbidities. The analysis model employed was Ordinal Logistic Regression, with the “bootstrap” resampling method used to enhance the confidence intervals. Statistical significance was set at *p* <0.05.

**Result:**

In total, 370 older people were screened, with 69.9 years old (± 6.5 years), of which 230 (42.6%) were female. In addition, 38,5% had high blood pressure, 25,5% had high capillary glycemia, and 23,3% had frailty. The prevalence of dementia was 6.75%. The adjusted ordinal regression model was significant (*p* = 0,00; pseudo R^2^=0,15). Among the variables significantly associated with prevalence of dementia, education was inversely associated (OR 0.45 (95% CI 0.30;0.68; *p* <0.01), altered capillary glycemia (OR 20.18 (95% CI 1.06;4.48; *p* = 0.03) and frailty was positively associated (OR 2031) (95% CI 1.44; 3.69; *p* <0.01).

**Conclusion:**

The prevalence of dementia among older primary health care users aligns with global rates, with factors such as education, altered capillary glycemia, and frailty playing significant roles. These findings are consistent with existing scientific literature on the topic.

